# A Pilot Study of Parameter-Optimized Low-Intensity Pulsed Ultrasound Stimulation for the Bone Marrow Mesenchymal Stem Cells Viability Improvement

**DOI:** 10.1155/2019/8386024

**Published:** 2019-10-01

**Authors:** Xiuzhi Yang, Yu Wu, Jiqing Li, Wuliang Yin, Yang An, Yanfen Wang, Man Wang, Qiuli Wu, Zhigang Qu, Guangzhi Ning, Shiqing Feng

**Affiliations:** ^1^College of Electronic Information and Automation, Tianjin University of Science & Technology, Dagu South Road No. 10308, Hexi District, Tianjin 300222, China; ^2^Advanced Structural Integrity International Joint Research Centre, Tianjin University of Science and Technology, Dagu South Road No. 1038, Hexi District, Tianjin 300222, China; ^3^School of Electronic and Electrical Engineering, Zhaoqing University, Zhaoqing Road, Duanzhou District, Zhaoqing 526061, Guangdong Province, China; ^4^Department of Orthopaedic, Tianjin Medical University General Hospital, Anshan Road No. 154, Heping District, Tianjin 300052, China; ^5^School of Electronic and Electrical Engineering, University of Manchester, Manchester M60 1QD, UK

## Abstract

To investigate how a back propagation neural network based on genetic algorithm (GA-BPNN) optimizes the low-intensity pulsed ultrasound (LIPUS) stimulation parameters to improve the bone marrow mesenchymal stem cells (BMSCs) viability further. The LIPUS parameters were set at various frequencies (0.6, 0.8, 1.0, and 1.2 MHz), voltages (5, 6, 7, and 8 V), and stimulation durations (3, 6, and 9 minutes). As only some discrete points can be set up in the experiments, the optimal LIPUS stimulation parameter may not be in the value of these settings. The GA-BPNN algorithm is used to optimize parameters of LIPUS to increase the BMSCs viability further. The BMSCs viability of the LIPUS-treated group was improved up to 19.57% (*P* < 0.01). With the optimization via the GA-BPNN algorithm, the viability of BMSCs was further improved by about 5.36% (*P* < 0.01) under the optimized condition of 6.92 V, 1.02 MHz, and 7.3 min. LIPUS is able to improve the BMSCs viability, which can be improved further by LIPUS with parameter optimization via GA-BPNN algorithm.

## 1. Introduction

Since the concept of tissue engineering and regenerative medicine has been proposed, as a particular type of MSCs, bone marrow mesenchymal stem cells (BMSCs) have broad application prospects in the field of cell transplantation. Studies have shown that BMSCs has strong proliferation and low immunological properties [1, 2] and gradually become the best source of seed cells in tissue engineering.

Low-intensity pulsed ultrasound (LIPUS) is an effective, noninvasive, and safe ultrasonic therapy [3–5]. Some animal experiments have proved that LIPUS can improve tissue regeneration [6–9]. At present, LIPUS parameters can be set to stimulate cells, but can only be set to some fixed and discrete voltages, frequencies, and stimulation durations. These settings may not be the optimal LIPUS stimulation parameters. The relationship between LIPUS parameters and BMSCs viability is complex and nonlinear, and therefore it is important to develop techniques to take these factors into account. GA-BPNN is capable of nonlinear multivariate data analysis and forecasting because of its ability to account for nonlinearity. It has been used in optimizing parameters in many fields such as manufacturing industry, electric power industry, and so forth [10–13].

The current study is a preliminary investigation on the use of GA-BPNN for further improvement of BMSCs viability. This method may help to optimize the parameters of LIPUS technique for cell transplantation.

## 2. Materials and Methods

### 2.1. BMSCs Culture

BMSCs were isolated from fifty female Wistar rats aged 4 weeks and weighing 180 ± 20 g. The Experimental Animal Ethics Committee of Tianjin Medical University approved all of the animal experimental protocols. The femurs and tibias of the rats were bluntly dissected with all connective tissue cleaned. Then the bone marrow was exposed by cutting off both ends of the femur. The bone marrow was flushed out with fetal bovine serum (FBS) (10%, v/v, Solarbio Co., Beijing, China) and cultured in LG-DMEM (Gibco, a brand of Thermo Fisher Scientific, Waltham, MA, USA), which was supplemented with fetal bovine serum (FBS) (10%, v/v, Solarbio Co., Beijing, China), streptomycin (100 *μ*L/100 mL, Solarbio Co., Beijing, China), and penicillin (100 *μ*L/100 mL, Solarbio Co., Beijing, China) in an incubator with 5% CO_2_ at 37°C. BMSCs were passaged when the cells reached about 85%–90% confluence. BMSCs at passage 3 were used for the LIPUS stimulation experiments.

### 2.2. Experimental Equipment

**Figure 1:**
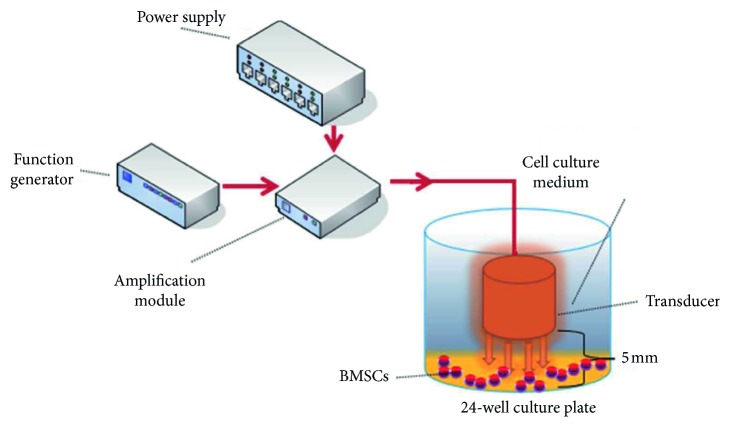
The LIPUS experimental equipment.

The schematic representation of LIPUS exposure setup is as below (Figure 1). The system basically consists of a power supply (HT2332, Henki), a function generator (AFG 3052C, Tektronix), an amplification module (THS4062, Texas Instruments), and a transducer (Shanghai XieMing Ultrasonic Equipment Co., Ltd). The central frequency of the transducer is 1 MHz, whose outside diameter is 10 mm. The probe is inserted into the cell culture dish to stimulate the cell, and the distance from the top of the transducer to the bottom of the culture plate is about 5 mm. At various voltages and frequencies, ultrasonic waves with different particular acoustic intensity were used to stimulate BMSCs (Table 1). The acoustic intensity was measured by Hangzhou Applied Acoustics Research Institute.

### 2.3. LIPUS Stimulation

BMSCs at passage 3 were used for the LIPUS stimulation experiments in a super-clean bench (Suzhou purification equipment Co., Ltd., Jiangsu, China). BMSCs were seeded onto Petri dishes at 1 × 10^4^ cell concentration in each Petri dish. The LIPUS-treated group contained 48 groups, and each group of experiments was repeated 20 times, thus 960 sets of data were collected. Prior to ultrasound exposure, the medium was washed three times with phosphate-buffered saline (PBS) (Solarbio Co., Beijing, China). LIPUS stimulates BMSCs after adding 1 ml of medium to each well. To determine the optimal LIPUS parameters, various voltages (5, 6, 7, and 8 V), frequencies (0.6, 0.8, 1, and 1.2 MHz), and stimulation durations (3, 6, and 9 min) were performed in the experiments. The control group underwent the same submersion but without ultrasound stimulation (0 MHz).

### 2.4. Evaluation of Cell Proliferation

Cell proliferation viability was measured by the cell counting kit-8 (CCK-8) according to the manufacturer's protocol (BestBio, China). After LIPUS stimulation, the cells were digested with 0.25% trypsin‐EDTA solution (Solarbio Co., Beijing, China). Subsequently, BMSCs were seeded in the 96-well culture plates at a density of 5 × 10^4^ cells/well (100 *μ*L) and cultured in the incubator for 24 h to adhere. 10 *μ*L CCK-8 solution was mixed carefully and then added into each well, and the plates were incubated for 3 h to evaluate cell proliferation viability. The absorbance at 450 nm was measured by a multifunctional plate reader (Varioskan Flash), and the OD values were recorded. All the absorbance rates are expressed as percent of the absorbance rate of the control group (without ultrasound stimulation, 0 MHz), which was set as 100%.

### 2.5. GA-BPNN Model

**Figure 2:**
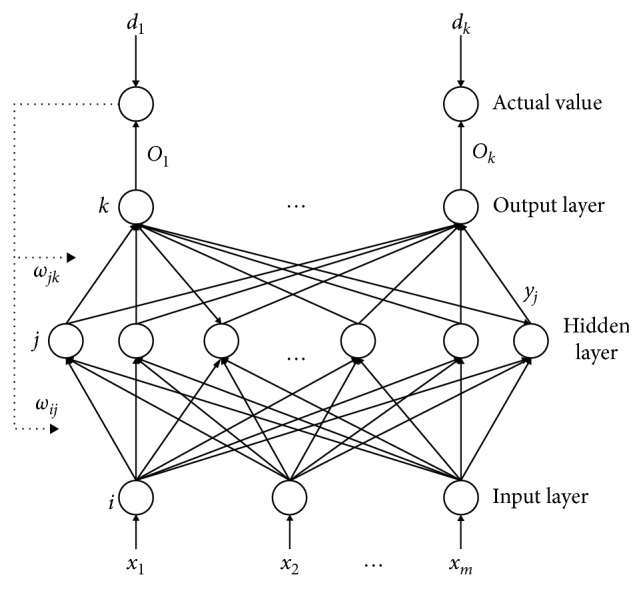
Structure diagram of the three-layer BPNN model.

BPNN is able to approach a nonlinear continuous function reasonably in theory [14]. BPNN is used to optimize the parameters of LIPUS to improve the viability of BMSCs further. BPNN consists of three layers: the input layer, the hidden layer, and the output layer (Figure 2). *x* is the input of BPNN, *d* is the output of BPNN, and *ω* is the neural network weights. BPNN learns by a rule, and then a corresponding decision is made. BPNN needs a certain amount of historical data, and then the network can learn the implicit knowledge in the data. The output error is used to estimate the error of the previous layer. According to the prediction error, the weights and thresholds of BPNN are able to be adjusted [11, 15, 16] so that the output of the BPNN is expected to approach the desired output.

The general weight adjustment formula of BPNN is as follows:

(1) $$\begin{array}{c} \left\{\begin{array}{c} \Delta \omega_{jk}=-\eta \frac{\partial E}{\partial \omega_{jk}}=\eta e_{k}y_{j}=\eta \left(d_{k}-O_{k}\right)y_{j}f^{\prime }\left({\text{net}}_{k}\right),\\ \Delta \omega_{ij}=-\eta \frac{\partial E}{\partial \omega_{ij}}=\eta e_{j}y_{i}=\eta \left(\overset{1}{\underset{k=1}{\sum }}e_{k}\omega_{jk}\right)x_{i}f^{\prime }\left({\text{net}}_{j}\right), \end{array}\right. \end{array}$$

where *ω* is network weight, Δ*ω* is weight increment, *E* is the error function of the output node of neural network: *E*=1/2∑_*K*=1_^*L*^(*d*_*k*_ − *O*_*k*_)^2^, *e*_*k*_ is the error back propagation signal from outer layers to inner ones, *L* is the number of output neurons, *f*′(net_*k*_) and *f*′(net_*j*_) are the derivatives of transfer function of output and hidden layer, the negative sign expresses the gradient descent, and the constant *η* ∈ (0,1) is the learning rate of network.

The output of hidden layer is calculated as follows:

(2) $$\begin{array}{c} y_{j}=f\left(\overset{n}{\underset{i=1}{\sum }}\omega_{ij}x_{i}-a_{j}\right),\;j=1,2,\ldots ,l, \end{array}$$

where *y*_*j*_ and *f* is the output of hidden layer and the incentive function of neurons, *j* is the neuron number of hidden layer, *m* is the neuron number of input layer, *ω*_*ij*_ is the weight factor between input layer and hidden layer, and *a*_*j*_ is the threshold value. Then, the predicting value of the output layer is calculated as follows:

(3) $$\begin{array}{c} O_{k}=\overset{l}{\underset{j=1}{\sum }}y_{j}\omega_{jk}-a_{k},\;k=1,2,\ldots ,n, \end{array}$$

where *ω*_*jk*_ is the weight factor between input layer and hidden layer and *k* is the neuron number of output layer.

**Figure 3:**
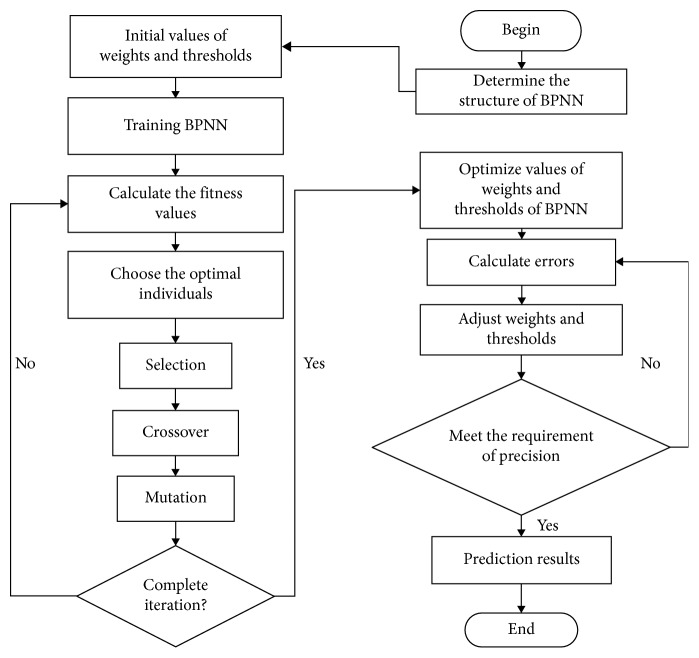
Flow chart of the GA-BPNN algorithm.

BPNN is one of the most widely used artificial neural networks. However, local optimization and overfitting are ineluctable in the BPNN calculation process. Genetic algorithm (GA) is a parallel stochastic search optimization method. BPNN had been improved by introducing GA, therefore, the whole algorithm is called GA-BPNN (Figure 3). GA-BPNN performs better than BPNN in terms of mean error, mean square error, and error probability. The weights and thresholds of BPNN are initialized, and then the network is trained.

GA-BPNN is a neural computation method and can effectively realize the nonlinear mapping of the input space to the output space. The three parameters (voltage, frequency, and simulation duration) are treated as the input of GA-BPNN, and the BMSCs viability is used as the output. By training these data, GA-BPNN is able to derive the main characteristics of these samples, and the optimal value can be obtained. After obtaining the optimal value, the verification experiments are needed to check the result. The optimal LIPUS parameters combining voltage, frequency, and stimulation duration, which is able to improve BMSCs viability further, can be used to stimulate BMSCs again.

### 2.6. Statistical Analysis

All statistical analyses were expressed as mean ± standard deviation (SD). Differences between the groups were compared using one-way analysis of variance (ANOVA) to determine the effects of voltage, frequency, and stimulation duration. A level of *P* < 0.05 value was considered statistically significant.

## 3. Results

### 3.1. BMSCs Viability Analysis

**Figure 4:**
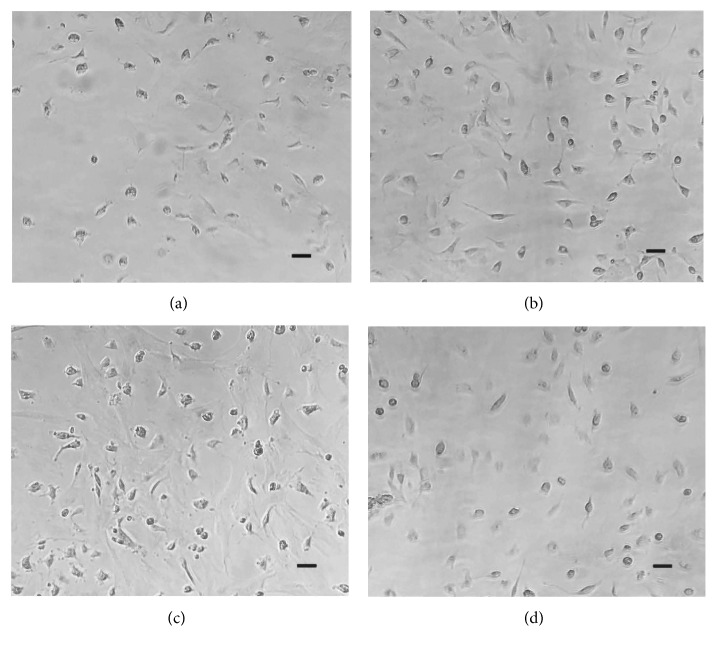
BMSCs proliferation with different parameters (*n* = 20). (a) BMSCs proliferation in the control group. (b) BMSCs proliferation with 3 min LIPUS stimulation. (c) BMSCs proliferation with 6 min LIPUS stimulation. (d) BMSCs proliferation with 9 min LIPUS stimulation (bar = 200 *μ*m).

The 3 passages of BMSCs were successfully obtained by culture, isolation, and purification. After being sterilized by 75% medical alcohol, the transducer was inserted into the culture medium. The LIPUS parameters were set at various frequencies (0.6, 0.8, 1.0, and 1.2 MHz), voltages (5, 6, 7, and 8 V), and stimulation durations (3, 6, and 9 minutes). Besides, the ultrasound frequency of the control group was set to 0 MHz. After 24 h, the BMSCs of the stimulation group and the control group were removed from the medium. The BMSCs were flushed and cultured in L-DMEM supplemented with 10% FBS, and then 10 *μ*L CCK-8 was added per culture medium. Before LIPUS stimulation, under the inverted microscope, the morphology of the cells was spherical, and they varied in size under DMIL LED inverted microscope (Leica Instrument Manufacturing Co., Ltd.). Compared with the control group, the counts of BMSCs stimulated by LIPUS are significantly increased. Besides, through LIPUS stimulation, the proliferation and morphology of BMSCs are different for different parameter combinations (voltage, frequency, and stimulation duration) (Figure 4).

**Figure 5:**
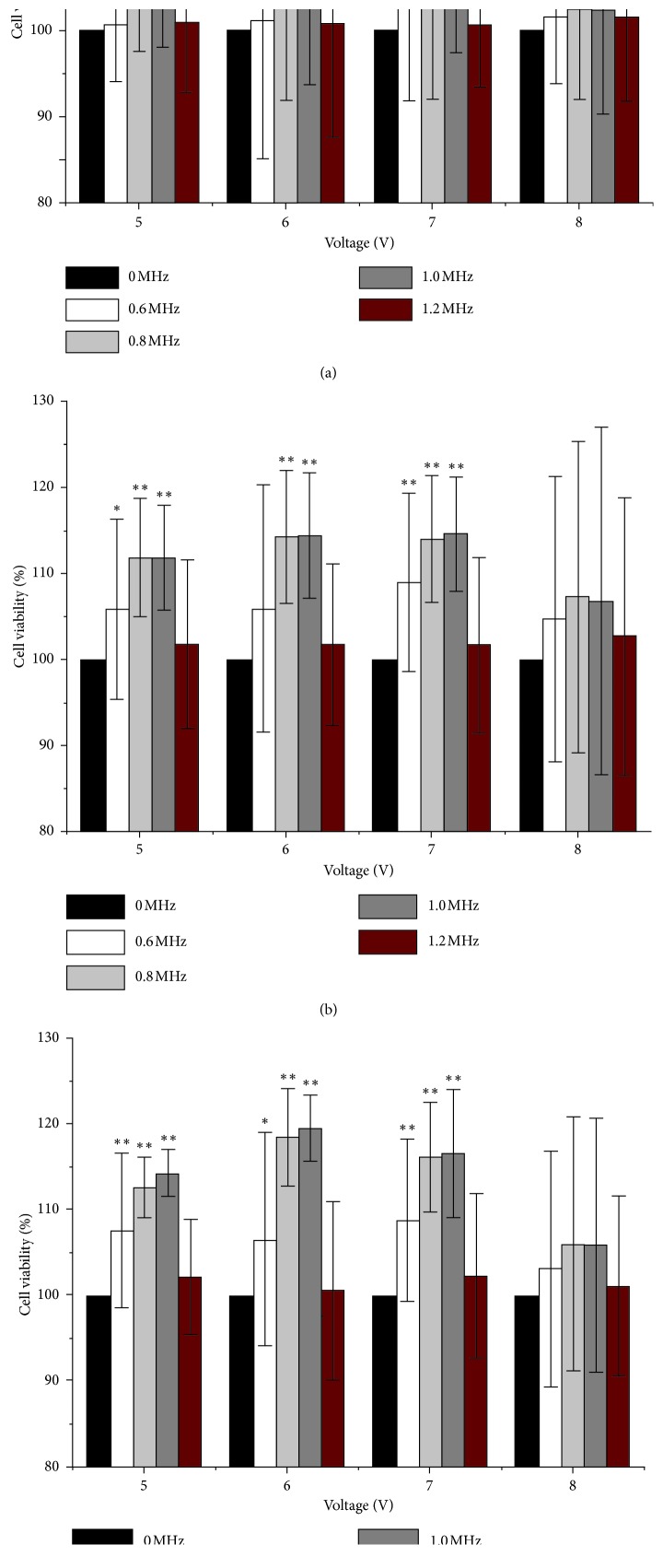
The BMSCs viability (%) after LIPUS stimulation with different parameters (voltage, frequency, and stimulation duration). *n* = 5 for each group. LIPUS-treated group (0.6, 0.8, 1, and 1.2 MHz) versus control group (0 MHz), one-way ANOVA, ^*∗*^*P* < 0.05, ^*∗∗*^*P* < 0.01. (a) The BMSCs viability varied with frequency and voltage when the stimulation duration was 3 min. (b) The BMSCs viability varied with frequency and voltage when the stimulation duration was 6 min. (c) The BMSCs viability varied with frequency and voltage when the stimulation duration was 9 min.

In order to investigate how LIPUS parameters influence the BMSCs viability, BMSCs were stimulated by various voltage, frequency, and stimulation duration. The BMSCs viability is different from various voltages and frequencies for different stimulation durations (Figure 5) (detailed data are provided in Tables 2–4). When the voltage is 6 V, the frequency is 1 MHz, and the stimulation duration is 9 min, the BMSCs viability is the strongest.

**Figure 6:**
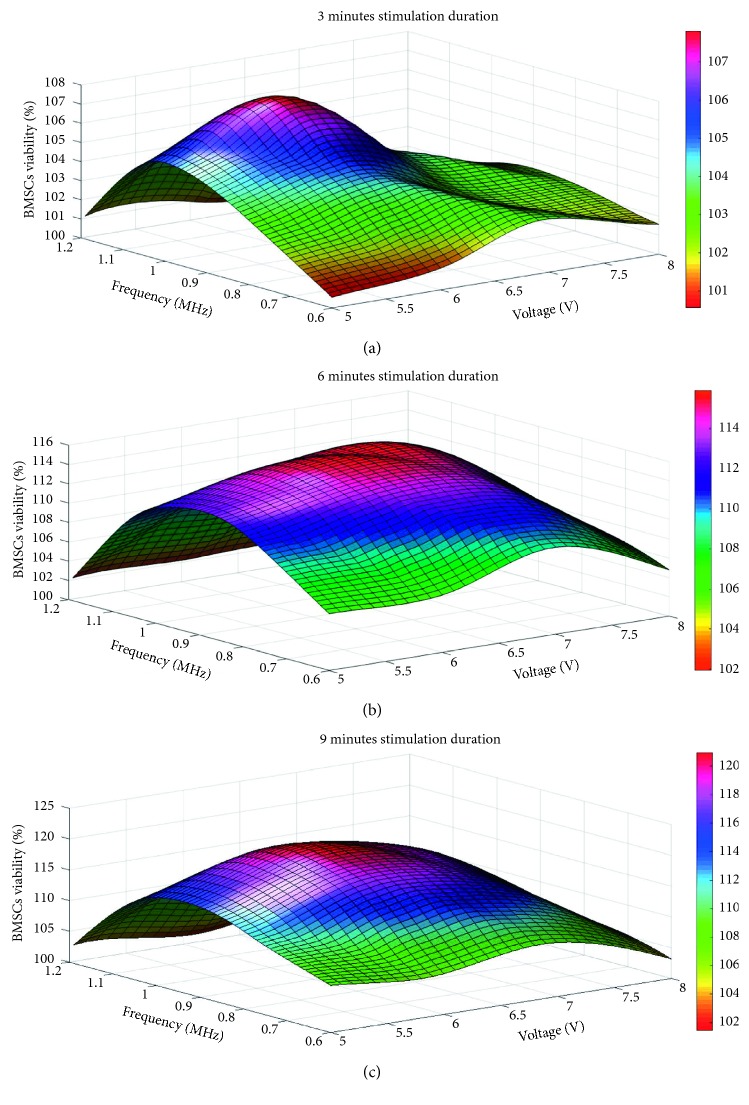
(a) The BMSCs viability with 3 min stimulation duration. (b) The BMSCs viability with 6 min stimulation duration. (c) The BMSCs viability with 9 min stimulation duration.

Colors represent BMSCs viability, and it is clear that the relationship between the 3 parameters (voltage, frequency, and stimulation duration) and BMSCs viability is complex and nonlinear (Figure 6). BMSCs viability is different with different voltage, frequency, and stimulation duration. Also the conditions in the experiments are likely not to contain the optimal LIPUS stimulation parameters, which is to be identified by the GA-BPNN algorithm developed in this work.

### 3.2. Application of GA-BPNN Model

There are 3 nodes, 8 nodes, and 2 nodes in the input layer, the hidden layer, and the output layer, respectively. The specified parameters of BPNN and GA were set up using the values given in Tables 5 and 6.

**Figure 7:**
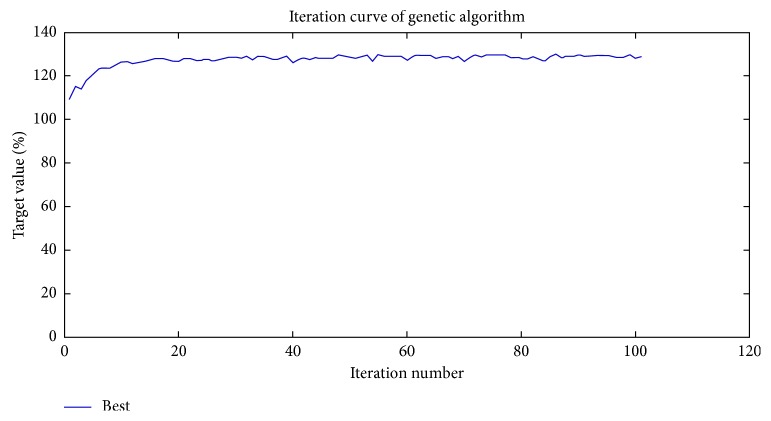
The prediction results of the GA-BPNN.

When the number of iteration increases over around 40, the curve of GA becomes stable and then reaches a plateau (Figure 7). The optimal combination of parameters can be achieved by the GA-BPNN, which are 6.92 V, 1.02 MHz, and 7.3 min, respectively.

### 3.3. Verification Experiments

**Figure 8:**
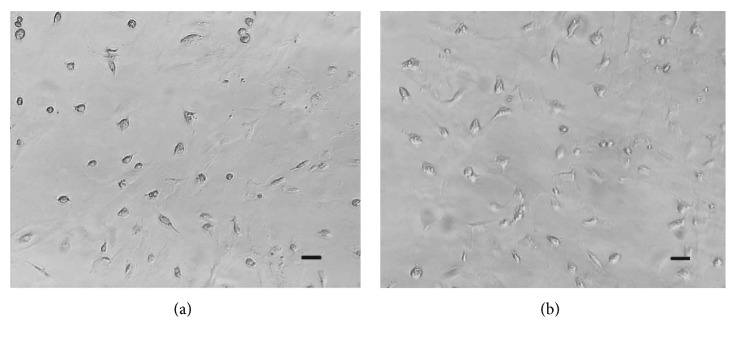
The verification experiments. (a) The control group. (b) The LIPUS-treated group under the optimized condition (6.92 V voltage, 1.02 MHz frequency, and 7.3 min stimulation duration) by GA-BPNN algorithm. *n* = 5 for each group; bar = 200 *μ*m.

It shows the BMSCs proliferation and morphology at validation experiments (Figure 8). The viability (124.93%) of BMSCs under the optimized condition (6.92 V, 1.02 MHz, and 7.3 min) via GA-BPNN algorithm is about 5.36% higher than that of LIPUS-treated group without the optimization (6 V, 1 MHz, and 9 min).

**Figure 9:**
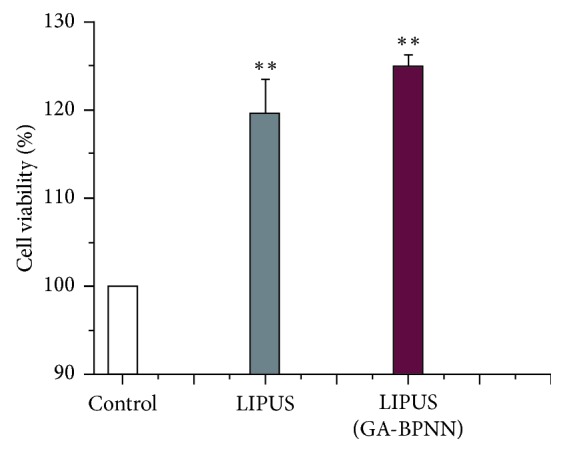
The BMSCs viability with different methods. Data presented as mean ± SD. ^*∗∗*^*P* < 0.01.

Compared with the control group, LIPUS stimulation is able to improve the viability of BMSCs (Figure 9 and Table 7). More importantly it should be noted that the BMSCs viability increased further after the GA-BPNN optimization.

## 4. Discussion

MSCs from a mesoderm origin are very attractive stem cells in the field of cell-based tissue regeneration and gene therapy [2, 17]. It is a good way to use stem cells to replace damaged tissue. However, the proliferation and differentiation of BMSCs need certain condition control. It would need a long exploration stage on how to avoid the potential risk factors such as tumor when BMSCs differentiate and proliferate in a certain way. BMSCs are thought to be multipotent cells. With certain stimulation, BMSCs can differentiate into bone cells, cartilage cells, muscle cells, fat cells, and so on [18]. BMSCs are not only able to secrete a variety of nerve growth factor but also able to promote the secretion of the central nervous system growth factor. Besides, BMSCs are able to promote local angiogenesis and vascular remodeling [19].

Studies [20–22] demonstrated that LIPUS is able to effectively promote fracture healing, fracture delayed tissue healing, and other bone defect regeneration. However, in the prior studies, the parameter setting of LIPUS on BMSCs is not optimized. In this work, GA-BPNN is chosen to preliminarily optimize three LIPUS parameters.

The artificial neural network can realize some functions on the basis of the understanding of the human brain neural network. GA-BPNN is a mathematical model of the human brain neural network and can be simulated by computer software. Besides, it is able to acquire knowledge by learning and store it on interconnected weights rather than in a specific storage unit. Corresponding to the human brain's receiving information, processing information, and making judgments, GA-BPNN formed the corresponding information processing model (input layer, hidden layer, and output layer). The relationship between LIPUS parameters and BMSCs viability is nonlinear. Fortunately, GA-BPNN, which is usually applied to simulate irregular nonlinear systems, is able to deal with the aforementioned case.

GA-BPNN was employed to find the optimal parameter combination of LIPUS stimulation, which provides a valuable reference for further fundamental and clinical research about the optimal treatment protocols for LIPUS. Compared with the control group, whose BMSCs viability is 100%, the BMSCs viability (119.57%) of the LIPUS-treated group was improved up to 19.57%. The viability of BMSCs (124.93%) was improved further by 5.36% by using the optimized parameters of LIPUS obtained by the GA-BPNN algorithm. The experimental data verified that the algorithm based on the GA-BPNN was able to optimize parameters of LIPUS to increase the BMSCs viability further.

It is important to recognize that this study presented some preliminary results and further work has been planned and carried out already. Firstly, only BMSCs were investigated in the current cells study. In fact, more cell studies should be performed to study the impacts of frequency, voltage, and stimulation of LIPUS treatment. Secondly, there were some differences in BMSCs between rats and humans. Thus, the results of this study might not be simply translated to the treatment of humans.

This study shows that the use of the GA-BPNN increases the viability of BMSCs. Therefore, further studies focusing on BMSCs transplantation in vivo are promising. In addition, the findings of this study may provide some meaningful research foundations for future clinical and basic research in medicine.

## 5. Conclusion

In this study, LIPUS is able to improve the BMSCs viability, which can be improved further by LIPUS with parameter optimization via GA-BPNN algorithm. The findings of this study provide a meaningful research foundation for future clinical and basic research in medicine.

## Figures and Tables

**Table 1:** Acoustic intensity (mW·cm^−2^) stimulates BMSCs at 5 mm.

Voltage (V)	Frequency (MHz)			
	0.6	0.8	1	1.2
5	13.9	3.4	33.7	3.7
6	19.3	4.7	48.2	5.1
7	28.9	6.8	53.9	8
8	37.3	8.5	69.3	10

**Table 2:** BMSCs viability (%) after LIPUS stimulation with 3 min.

Voltage (V)	Frequency (MHz)				
	0	0.6	0.8	1	1.2
5	100	100.57 ± 6.52	103.40 ± 5.91^*∗*^	105.17 ± 7.17^*∗∗*^	100.92 ± 8.18
Confidence intervals (%)	[100,100]	[97.52,103.62]	[100.63,106.16]	[101.81,108.53]	[97.09,104.74]
*P* values	—	0.699	0.014	0.003	0.619

6	100	101.15 ± 16.08	104.81 ± 12.95	107.72 ± 14.07^*∗*^	100.71 ± 13.13
Confidence intervals (%)	[100,100]	[93.62,108.68]	[98.75,110.87]	[101.13,114.30]	[94.57,106.86]
*P* values	—	0.751	0.105	0.019	0.81

7	100	102.8 ± 10.92	102.84 ± 10.81	103.94 ± 6.54^*∗*^	100.59 ± 7.15
Confidence intervals (%)	[100,100]	[97.69,107.91]	[97.78,107.90]	[100.88,106.10]	[97.24,103.94]
*P* values	—	0.259	0.248	0.01	0.715

8	100	101.57 ± 7.73	102.46 ± 10.56	102.34 ± 12.14	101.48 ± 9.82
Confidence intervals (%)	[100,100]	[97.95,105.20]	[97.51,107.40]	[96.66,108.02]	[96.88,106.07]
*P* values	—	0.368	0.305	0.394	0.505

Data presented as mean ± SD, *n* = 20 (^*∗*^*P* < 0.05, ^*∗∗*^*P* < 0.01).

**Table 3:** BMSCs viability (%) after LIPUS stimulation with 6 min.

Voltage (V)	Frequency (MHz)				
	0	0.6	0.8	1	1.2
5	100	105.89 ± 10.51^*∗*^	111.86 ± 6.87^*∗∗*^	111.91 ± 6.09^*∗∗*^	101.8 ± 9.8
Confidence intervals (%)	[100,100]	[100.97,110.81]	[108.64,115.07]	[109.06,114.76]	[97.21,106.38]
*P* values	—	0.017	2.64*E* − 09	1.22*E* − 10	0.418

6	100	105.94 ± 14.42	114.28 ± 7.7^*∗∗*^	114.45 ± 7.28^*∗∗*^	101.75 ± 9.43
Confidence intervals (%)	[100,100]	[99.19,112.69]	[110.68,117.89]	[111.04,117.86]	[97.33,106.16]
*P* values	—	0.073	4.70*E* − 10	8.56*E* − 11	0.412

7	100	109.02 ± 10.41^*∗∗*^	114.06 ± 7.33^*∗∗*^	114.64 ± 6.6^*∗∗*^	101.73 ± 10.3
Confidence intervals (%)	[100,100]	[104.15,113.90]	[110.63,117.49]	[111.55,117.73]	[96.91,106.55]
*P* values	—	0.0004	1.99*E* − 10	4.24*E* − 12	0.458

8	100	104.79 ± 16.66	107.28 ± 18.14	106.86 ± 20.24	102.77 ± 16.21
Confidence intervals (%)	[100,100]	[96.99,112.58]	[98.79,115.77]	[97.38,116.33]	[95.18,110.35]
*P* values	—	0.206	0.081	0.138	0.45

Data presented as mean ± SD, *n* = 20 (^*∗*^*P* < 0.05, ^*∗∗*^*P* < 0.01).

**Table 4:** BMSCs viability (%) after LIPUS stimulation with 9 min.

Voltage (V)	Frequency (MHz)				
	0	0.6	0.8	1	1.2
5	100	107.64 ± 8.97^*∗∗*^	112.64 ± 3.53^*∗∗*^	114.33 ± 2.74^*∗∗*^	102.18 ± 6.7
Confidence intervals (%)	[100,100]	[103.44,111.84]	[110.99,114.29]	[113.04,115.61]	[99.05,105.32]
*P* values	—	0.0005	1.66*E* − 18	3.90*E* − 24	0.153

6	100	106.63 ± 12.5^*∗*^	118.55 ± 5.75^*∗∗*^	119.57 ± 3.85^*∗∗*^	100.67 ± 10.46
Confidence intervals (%)	[100,100]	[100.78,112.49]	[115.87,121.24]	[117.77,121.37]	[95.77,105.56]
*P* values	—	0.023	5.15*E* − 17	1.00*E* − 23	0.777

7	100	108.78 ± 9.49^*∗∗*^	116.21 ± 6.41^*∗∗*^	116.61 ± 7.49^*∗∗*^	102.36 ± 9.62
Confidence intervals (%)	[100,100]	[104.34,113.22]	[113.21,119.21]	[113.10,120.11]	[97.85,106.86]
*P* values	—	0.0002	9.86*E* − 14	4.28*E* − 12	0.28

8	100	103.17 ± 13.78	106.05 ± 14.84	105.95 ± 14.88	101.17 ± 10.47
Confidence intervals (%)	[100,100]	[96.72,109.62]	[99.10,112.10]	[98.99,112.91]	[96.27,106.07]
*P* values	—	0.31	0.076	0.082	0.62

Data presented as mean ± SD, *n* = 20 (^*∗*^*P* < 0.05, ^*∗∗*^*P* < 0.01).

**Table 5:** The specified parameters of BPNN.

Name	Input	Output	Maximum number of epochs	Learning rate	Accuracy
Number	3	1	1000	0.1	0.0001

**Table 6:** The specified parameters of GA.

Name	Population size	Iteration	Mutation probability (PM)	Crossover probability (PC)
Number	50	100	0.1	0.8

**Table 7:** BMSCs viability (%) with different methods. Data presented as mean ± SD, *n* = 5. (^*∗∗*^*P* < 0.01).

	Control	LIPUS-treated	LIPUS-treated (GA-BPNN)
BMSCs viability (%)	100	119.57 ± 3.85^*∗∗*^	124.93 ± 1.28^*∗∗*^
Confidence intervals (%)	[100,100]	[116.6,122.07]	[123.34,126.52]
*P* values	—	4.73*E* − 08	8.65*E* − 11

## Data Availability

The data used to support the findings of this study are available from the corresponding author upon request.
